# What proportion of people who use cannabis in Germany have spoken with their general practitioner about their consumption? A repeated cross-sectional representative population survey

**DOI:** 10.1186/s42238-025-00329-0

**Published:** 2025-10-07

**Authors:** Benjamin Borchardt, Stephanie Klosterhalfen, Daniel Kotz

**Affiliations:** 1 Institute of General Practice, Medical Faculty, University of Duisburg-Essen, Hufelandstr. 55, Essen, 45122 Germany; 2 Institute of General Practice, Addiction Research and Clinical Epidemiology Unit, Medical Faculty and University Hospital Düsseldorf, Heinrich Heine University Düsseldorf, Düsseldorf, Germany; 3 Department of Behavioural Science and Health, University College London, London, UK

**Keywords:** Cannabis, Primary care, General practice, Family medicine, Cannabis legalisation, Consultation

## Abstract

**Introduction:**

On April 1st, 2024, Germany’s cannabis legalisation bill entered into force. It is unknown what effect the legal changes could have, if any, on the prevalence of cannabis use. Preventive measures of cannabis-related health impacts are best possible if harmful use is recognised and addressed at an early stage. Our aim was to estimate the proportion of people who used cannabis in Germany that discussed their cannabis consumption with their general practitioner (GP), on the initiative of either the GP or the patient, and how these varied according to person characteristics.

**Methods:**

We used data from the German Study on Tobacco Use (DEBRA). This is a repeated ongoing representative cross-sectional household survey on the use of tobacco and alternative nicotine delivery systems in Germany in people aged 14 years and older. In bi-monthly study waves we asked all respondents who stated that they had consumed cannabis before whether they had ever spoken with their GP about their cannabis use or received advice about it. We estimated the proportion including 95% confidence intervals and assessed possible associations with person characteristics using univariate logistic regression models.

**Results:**

Of 2,057 people who have ever used cannabis, 7.0% [95%CI = 5.9; 8.2%] (*n* = 139) reported having spoken to their general practitioner about their cannabis use or having sought advice in this regard. This response was associated with older age (65 +), low educational attainment, low income and frequent cannabis use. Conversation with a GP was also more common among people who had used cannabis in the past year (16.2%; 95%CI = 13.5; 19.6%).

**Conclusions:**

Around one in fifteen people who have used cannabis before has ever spoken with his or her GP, or received advice, about his or her cannabis consumption. In people with past-year and especially frequent use, the proportions are highest, ranging between 16 to 26%. The awareness of this topic among GPs needs to be increased. Furthermore, a future re-evaluation of what impact cannabis legalisation will have on the frequency of consultation about cannabis consumption in primary care is necessary.

## Introduction

As of April 1 st, 2024, the German parliament legalised cannabis possession and home cultivation (German Federal Ministry of Health 2024; German Federal Ministry of Health 2025). We recently showed that at least one in twenty residents in Germany consumed cannabis in the past year, with a higher prevalence among younger people (Kotz D, et al. 2024). Irrespective of the legal environment, consumption rates have been increasing for the past two to three decades, and are expected to rise further (Olderbak S, et al. 2023). Whether the legal change could have an impact on consumption rates is unknown. A clear effect of legalisation on consumption rates has not been shown yet due to mixed results (Pravosud V, et al. 2024; Drugs EMCf, et al. 2004; Caulkins JP. 2024).

Apart from decriminalising possession, the change in legislation also simplified the availability of cannabis. Non-profit organisations and private individuals are now allowed to cultivate cannabis. Furthermore, a second pillar envisages granting licensed shops the permission to sell cannabis as part of regional model projects, for a limited five-year period. This second pillar will be scientifically evaluated (German Federal Ministry of Health 2025; Federal Government of Germany 2024).

It was emphasised by the German government that it aims to strengthen the prevention of cannabis-related negative health impacts as part of the legalisation process. Harms of cannabis use include, for instance, an increased risk for psychosis with a dose-dependent relationship (Marconi A, et al. 2016). Impairment of cognitive functions is another relevant harm which has also been shown to affect educational outcomes in adolescents (Cyrus E, et al. 2021; Lorenzetti V, et al. 2020). Preventive measures regarding cannabis-related harms can be greatly facilitated by an early knowledge of problematic cannabis use by health professionals, and by patients consulting health professionals in case of problematic use. General practitioners (GPs) play a special role in this regard, as they are considered a person of trust and because they acknowledge a family medicine perspective. GPs are usually the first point of contact for many health problems (Nolting & Zich, 2021), and most people in Germany contact their GP at least once a year (Prütz F, et al. 2021). However, it is unknown what proportion of people who use or have used cannabis had previously consulted their GP due to their cannabis consumption, since to our knowledge this topic has not been investigated so far. At most, there are studies which have focused on the proportion of GPs who screen for cannabis use (Beck F, et al. 2011), or screening studies to determine the prevalence of cannabis use (Gelberg L, et al. 2024). A comparison of whether legalisation will, in fact, lead to an increase of health service utilisation can only be possible if a status quo is determined as baseline beforehand, ideally before the new law change enters into force. In addition, knowledge of which patients in general practice are more often and which less often involved in discussion of cannabis use could facilitate identification of those who should be focused on in particular, in whatever form, for example, by receiving more attention from their GP or through other interventions. Therefore, we strived to estimate the proportion of people in Germany who discussed their cannabis consumption with their GP among those who use or have used cannabis, on the initiative of either the GP or the patient. Although the legalisation affects only adults, the proportions are equally relevant for minors. Child and youth protection was explicitly addressed in the law and it cannot been ruled out that the legalisation might have an effect beyond the legally set age limit (German Federal Ministry of Health 2025). Using data from an ongoing representative population survey, we sought to answer the following two research questions:

1. In the general German population aged 14 years and older, what proportion of people who have ever used cannabis have ever spoken with or received advice from their GP about their cannabis use?
2. In the general German population aged 14 years and older, how does the proportion of people who have ever used cannabis and who have spoken with their GP about their cannabis use vary according to relevant sociodemographic characteristics, region of living and frequency of cannabis use?

## Methods

### Study population

We used data from the German Study on Tobacco Use (DEBRA: *Deutsche Befragung zum Rauchverhalten*), an ongoing representative cross-sectional household survey on tobacco use in Germany in people aged 14 years and older which commenced in 2016. The rationale of the DEBRA study has been published elsewhere (Kastaun S, et al. 2017). In short, 2000 people aged 14 years and older are interviewed on the use of tobacco and alternative nicotine delivery products every other month in a new representative sample using computer-assisted face-to-face household interviews. The general items are openly accessible online (https://osf.io/snm3p). Since wave 22 (January 2020), respondents in the DEBRA study have been selected by using a dual frame design: a composition of random stratified sampling (50% of the sample) and quota sampling (50% of the sample; see also https://osf.io/s2wxc).

During five waves of the DEBRA study (March to November 2023), we included two additional questions on cannabis use. Since this survey was conducted before the law entered into force, the topic was introduced on the basis of information about legislative changes that were planned but not yet implemented at the time. It was also specified that products without drug-like exhilarating effects, such as products with cannabidiol, were not meant. Furthermore, participants were assured once more that their responses were anonymous since cannabis use and possession was still illegal in Germany at the time of the interviews. For details see our a priori published study protocol which also includes a detailed analysis plan: https://osf.io/gs4fd. All respondents who agreed to answer questions related to cannabis use were included in our analysis.

### Measurements

First, cannabis use was surveyed with the question “Have you ever consumed cannabis?” The response options provided were designed to narrow down the frequency of use. We categorised all respondents either into people who had ever used cannabis or who had never used cannabis. Furthermore, only the subgroup of people with past-year use (defined as consumption within the past 12 months) was further categorised into people with frequent (i.e. at least once a week) or non-frequent use (i.e. less than once a week). For details of the response options as well as the categorisation process see our study protocol (https://osf.io/gs4fd).

To measure the proportion of people who ever used cannabis who have ever spoken with, or received advice from, their GP about their cannabis use, we asked all people with ever use: “Have you ever spoken to your general practitioner about your cannabis use or sought advice in this regard?” The response options were as follows: 1. “Yes, I have ever spoken to my general practitioner about my cannabis use or sought advice in this regard”; 2. “No, I have never spoken to my general practitioner about my cannabis use or sought advice in this regard”; 3. “I do not have a general practitioner”; 4. “No response”.

The following person characteristics, with the respective categories shown in brackets, were collected and analysed as further variables: age (14–24, 25–39, 40–64, 65 + years), sex (male or female), educational attainment (low = junior high school equivalent or no qualification, middle = secondary school equivalent, high = high school equivalent or advanced technical college equivalent), migration background (one or both parents born abroad, none born abroad) and region of living (rural = < 20,000 inhabitants, urban = 20,000–500,000 inhabitants, metropolitan = > 500,000 inhabitants). Another variable was the monthly disposable household income per person in the household (low = < 1000 €, middle = ≥ 1000 € to < 2333 €, high = ≥ 2333 €). This variable was adapted using an equalisation technique (OECD-modified equivalence scale) of the Organisation for Economic Co-operation and Development (OECD) to account for different household sizes and compositions. The adjustment process is described in detail elsewhere (https://osf.io/387fg). An approximate 20–60–20% distribution defined the category boundaries of 1000 € and 2333 € for the variable monthly disposable household income per person.

### Statistical methods

For all analyses, we utilised complete cases only as well as weighted data to ensure a representative sample of the German population accounting for personal and household characteristics. Details of the weighting process can be found in the DEBRA study protocol (Kastaun S, et al. 2017). Data from the five waves of the DEBRA study, which included the questions on cannabis use, were aggregated. To answer our first research question, we estimated the proportion of people who ever used cannabis who have ever spoken with their GP, or received advice, about their cannabis use, with the 95% confidence interval. While not previously outlined in our study protocol, we additionally estimated the proportion for people who used cannabis during the past year. To answer our second research question, we estimated proportions and 95% confidence intervals among people who ever used cannabis who have ever spoken with their GP, or received advice, about their cannabis use, stratified by the person characteristics age, sex, educational attainment, income, migration background, region of living, and —in the subgroup of people who used cannabis during the past year— frequency of cannabis use. The denominator for all estimated proportions comprised all people who ever used cannabis including those who answered not having a GP (*n* = 146 unweighted) and those who did not respond to this question (*n* = 14 unweighted). In addition, using univariate logistic regression models we estimated to what extent the same person characteristics (independent variables) were associated with a conversation with, or advice from, the GP about cannabis use (yes vs. no; dependent variable) among people who ever used cannabis. We reported odds ratios with 95% confidence intervals to assess effect size and precision. In the regression analyses, those reporting not having a GP or who gave no response were excluded.

## Results

Among 10,588 people interviewed, 9,718 respondents (91.8%) agreed to answer the questions on cannabis. Of those, 2,057 (21.2%) ever used cannabis and, of this subset, 2,043 (99.3%) answered the question regarding a discussion with or advice from their GP (all n unweighted). Baseline characteristics of the cohort who had ever used cannabis are presented in Table 1. The sample had a mean age of 41.1 years (SD = 15.7), the majority were men (63.5%) and had no migration background (78.1%). High educational attainment (41.8%), middle income group (57.5%) and urban region of living (41.3%) were the most frequent characteristics. Past-year cannabis use was reported by 591 people, 29.6% of those with ever cannabis use.

**Table 1:** Sample characteristics of people who ever used cannabis (weighted data, *n* = 1,998)

**Characteristic**	
Age in years, mean (SD)	41.1 (15.7)
Sex	
Male	63.5 (1269)
Female	36.5 (728)
Educational attainment^a^	
Low	22.3 (432)
Middle	36.0 (697)
High	41.8 (810)
Income^b^	
Low (< 1,000€)	13.8 (275)
Middle (1,000–2,333€)	57.5 (1145)
High (≥ 2,333€ or higher)	28.7 (571)
Migration background	
Yes (one or both parents born abroad)	21.9 (429)
No	78.1 (1530)
Region of living^c^	
Rural (< 20,000 inhabitants)	31.9 (638)
Urban (20,000–500,000 inhabitants)	41.3 (826)
Metropolitan (> 500,000 inhabitants)	26.7 (534)
Frequency of cannabis use^d^	
Frequent (at least once a week)	27.8 (164)
Non-frequent (less than once a week)	72.2 (426)

Numbers present percentages (number), unless otherwise stated. Missing values vary from 0% (age, sex and region of living) up to 3.0% (educational attainment)

^a^Low = junior high school equivalent or no qualification, middle = secondary school equivalent, high = high school equivalent or advanced technical college equivalent

^b^Details: https://osf.io/387fg

^c^Details: https://osf.io/zp7c6

^d^In the subgroup of people with past-year use (*n* = 591)

Among those with ever cannabis use, 7.0% [95%CI = 5.9; 8.2%] (*n* = 139) answered: ‘Yes, I have ever spoken to my general practitioner about my cannabis use or sought advice about it.’ This proportion was higher in people aged 65 years or older (12.5%; 95% CI = 7.9; 18.5%), people with low educational attainment (12.3%; 95% CI = 9.3; 15.7%) and low income (14.2%; 95% CI = 10.3; 18.9%). Among the subsample of people with past-year cannabis use, people with frequent cannabis use (26.2%; 95% CI = 19.7; 33.6%) more often had a conversation with a GP than those with non-frequent use (12.4%; 95% CI = 9.4; 15.9%) (see Table 2). The overall proportion among people with past-year use was 16.2% (95% CI = 13.5; 19.6%) (data not shown in the table).

**Table 2:** Cannabis use discussed with GP by person characteristics among people who ever used cannabis – Proportions (weighted data, *n* = 1,998)

	**% [95% CI]**
Total	7.0 [5.9; 8.2]
Age in years	
14–24	5.0 [3.0; 8.0]
25–39	8.8 [6.8; 11.2]
40–64	5.2 [3.8; 7.0]
65 +	12.5 [7.9; 18.5]
Sex	
Male	7.4 [6.0; 9.0]
Female	6.2 [4.5; 8.2]
Educational attainment^a^	
Low	12.3 [9.3; 15.7]
Middle	4.9 [3.4; 6.8]
High	5.8 [4.3; 7.7]
Income^b^	
Low (< 1,000€)	14.2 [10.3; 18.9]
Middle (1,000–2,333€)	5.3 [4.1; 6.8]
High (≥ 2,333€ or higher)	7.0 [5.0; 9.4]
Migration background	
Yes (one or both parents born abroad)	6.5 [4.4; 9.3]
No	6.7 [5.5; 8.1]
Region of living^c^	
Rural (< 20,000 inhabitants)	6.9 [5.1; 9.2]
Urban (20,000–500,000 inhabitants)	6.9 [5.3; 8.8]
Metropolitan (> 500,000 inhabitants)	7.1 [5.1; 9.6]
Frequency of cannabis use^d^	
Non-frequent (less than once a week)	12.4 [9.4; 15.9]
Frequent (at least once a week)	26.2 [19.7; 33.6]

Percentages are presented within each stratum, e.g. 5.0% of 14- to 24-year-olds have ever spoken with their GP, or received advice, about their cannabis use. 95% CI = 95% confidence interval around percentage. Missing values vary from 0% (age) up to 3.0% (educational attainment)

^a^Low = junior high school equivalent or no qualification, middle = secondary school equivalent, high = high school equivalent or advanced technical college equivalent

^b^Details: https://osf.io/387fg

^c^Details: https://osf.io/zp7c6

^d^In the subgroup of people with past-year cannabis use (*n* = 591), among this subgroup overall 16.2% (95% CI = 13.5; 19.6%) had discussed their cannabis use with their GP

Our regression analyses confirmed the descriptive results. Conversation with, or advice from, the GP on cannabis use was associated with older age (65 +), low educational attainment, low income, and frequent cannabis use. Of the people who used cannabis, those with higher educational attainment and higher income had at least 59% lower odds of having had spoken with their GP, or received advice, about their consumption. People with frequent use had about 150% higher odds of having discussed cannabis use than those with non-frequent use (see Table 3).

**Table 3:** Cannabis use discussed with GP by person characteristics among people who ever used cannabis – Associations (weighted data, *n* = 1,841)

	**OR [95% CI]**
Age in years	
14–24 (ref)	1.00
25–39	1.73 [0.99; 3.02]
40–64	0.98 [0.55; 1.74]
65 +	2.38 [1.21; 4.66]
Sex	
Male (ref)	1.00
Female	0.79 [0.55; 1.14]
Educational attainment^a^	
Low (ref)	1.00
Middle	0.34 [0.22; 0.54]
High	0.41 [0.27; 0.62]
Income^b^	
Low (< 1,000€) (ref)	1.00
Middle (1,000–2,333€)	0.32 [0.21; 0.49]
High (≥ 2,333€ or higher)	0.41 [0.26; 0.65]
Migration background	
Yes (one or both parents born abroad) (ref)	1.00
No	1.00 [0.65; 1.54]
Region of living^c^	
Rural (< 20,000 inhabitants) (ref)	1.00
Urban (20,000–500,000 inhabitants)	0.98 [0.65; 1.48]
Metropolitan (> 500,000 inhabitants)	0.99 [0.63; 1.56]
Frequency of cannabis use^d^	
Non-frequent (less than once a week) (ref)	1.00
Frequent (at least once a week)	2.53 [1.60; 3.99]

Odds Ratios derived from separate logistic regression models. Missing values vary from 0% (age) up to 1.6% (migration background)

^a^Low = junior high school equivalent or no qualification, middle = secondary school equivalent, high = high school equivalent or advanced technical college equivalent

^b^Details: https://osf.io/387fg

^c^Details: https://osf.io/zp7c6

^d^In the subgroup of people with past-year cannabis use (*n* = 529)

## Discussion

Our results of a representative sample of the German population show that 6–8% of people who ever used cannabis have discussed their consumption with their GP at some point. The proportion is higher in people with past-year use, particularly with frequent use as well as in low income and low education groups.

To our knowledge, this is the very first study to look at GP consultation on cannabis use in Germany. Studies from other countries are rare and, at most, focused on screening for cannabis use (Beck F, et al. 2011; Gelberg L, et al. 2024). This is suprising since one goal of primary care should be the prevention of health conditions related to non-medical substance use, of which screening followed by a brief intervention would be an integral part (Strobbe S, 2014). We were able to characterise the situation in Germany before the law on cannabis legalisation became effective. An interpretation of our results in relative terms is challenging since comparative figures are lacking. The fact that fewer than one in every ten persons with cannabis use has spoken with their GP about their use appears sparse on first impression. However, ever cannabis use also included those who had merely tried cannabis a few times and then stopped altogether. On the other hand, we did not differentiate between non-medical use and the use of medically prescribed cannabis. Medically prescribed cannabis is legal in Germany (Abuhasira R, et al. 2018), and these users could have influenced the figures upwards. Negative health effects of cannabis consumption are dosage dependent (Marconi A, et al. 2016). In people with past-year and especially frequent use we found much higher numbers of people who had talked with their GP about their consumption. One in four people with frequent use (i.e. weekly) reported a discussion with their GP about their consumption. In absolute terms this number is more promising. No clear treshold exists as to what proportion should be aspired to in primary care. One could argue that it should be the goal of health professionals to address the use of harmul substances with everyone who uses them, without exception, or at least with those who see their GP regularly. This can only be achieved with rigorous screening and has not been accomplished for smoking or alcohol consumption either. Cogordan et al. reported that, in France, 36.7% of patients in general practice reported having talked to their GP about tobacco smoking and 16.8% about alcohol consumption. (Cogordan C, et al. 2020)To our knowledge, estimates regarding other substances like opiates or other illegal substances are not available. In Germany, while GPs usually do not systematically screen for substance use, as part of the repeatedly performed (up to every three years in people over 35 years of age) general health examination they are instructed to enquire about tobacco smoking and alcohol, but not explicitly about other substances (Federal Joint Committee of Germany 2021). Another analysis of data from the DEBRA study showed that during the last visit of tobacco smokers with their GP in Germany, smoking was part of the conversation content in 25.7% of cases, with advice to stop smoking in 17.6% of cases (Kastaun S, et al. 2019). Around 30% of people with past-year cannabis use develop cannabis use disorder, which means they meet the DSM-IV (Diagnostic and Statistical Manual of Mental Disorders, fourth edition) criteria for either abuse or dependence (Hasin DS, et al. 2015). One could argue that these mainly present the people who should be identified by their GPs followed by further advice on this matter. Our findings show that this proportion is not reached. Screening every single person might not be reasonable, but GPs clearly need a strategy to be able to address a greater proportion of their patients, especially those with frequent cannabis use and, in particular, younger people. The willingness of patients in general, in particular younger ones, must also be taken into account (Fortin F, et al. 2022), as well as what follows consultation. Effective interventions which could be offered in general practice are needed (Laporte C, et al. 2017).

Since our analysis was not designed to identify causal mechanisms which led to a conversation with a GP, but rather to identify subgroups with a greater need for attention, we can only speculate about why a discussion about cannabis use was more common in lower socio-demographic groups and in older patients. We know that a similar pattern regarding alcohol consumption exists (Kastaun S, et al. 2022). People from higher socio-demographic groups might find it harder to admit consumption due to stigmatisation, or GPs might expect use more often in lower socio-demographic groups. The frequency of actually visiting one’s GP is likely to have an influence as well. Older people tend to have more comorbidities and therefore see their GP more often (Prütz F, et al. 2021; Babitsch B, et al. 2012), meaning that more chances arise for discussing other health topics. A request to be prescribed medical cannabis could at least partially explain the higher proportion of older people having discussed cannabis consumption (Matson TE, et al. 2021). An association with frequency of cannabis use lets us suspect that people with frequent cannabis use possibly experience some level of suffering or that they might be more easy identifiable by physicians due to certain personality traits (Chabrol H, et al. 2015).

One assumption or argument to legalise cannabis is that people who use cannabis will feel more comfortable to address this topic in conversations with health professionals since they will not need to admit illegal behaviour anymore (Manthey J, et al. 2024). It remains to be seen whether cannabis use will be discussed more frequently during GP consultations after legalisation in Germany. Even if this increases the number of people with cannabis use who consult their GP, the percentage will most likely remain low. Therefore, our results highlight the importance of preventive measures for cannabis-related negative health impacts, especially in primary care.

Our findings may have a number of important implications for daily practice in primary care. To begin with, the awareness of this topic among GPs has to be increased. Cannabis consumption is not a rare phenomenon (Kotz D, et al. 2024; Lapham GT, et al. 2017). A change in practice is required here, alongside training GPs on how to advise people with cannabis use and detect cannabis-related health problems early, especially in younger people (Fischer B, et al. 2022). GPs should also focus more on patients in the middle and high educational attainment and income groups, although it is equally possible that our results are due to these people talking about their consumption on their own initiative less often.

### Strengths and limitations

Our study has several limitations which need to be adressed. Our results did not allow us to distinguish whether patients or GPs initiated the conversation and did not differ between medically prescribed vs. non-medical cannabis use. Furthermore, we were not able to identify why cannabis use was a subject of discussion, for instance, to what extent symptoms of cannabis-related negative health impacts were the reason or if GPs screened for cannabis use. To a relevant extent, minors do not see a GP for healthcare provision, but instead a paediatrician who serves as a GP. The results for the age category of 14- to 24-year-olds might therefore underestimate the proportion. Other limitations of our analysis are the use of self-reported data and therefore a possible under-reporting of cannabis use as discussed in a previous publication (Kotz D, et al. 2024), as well as possible recall bias. We did not control for confounding in our univarite regression analyses. Therefore, we cannot make assumptions about direct relationships; instead, our independent variables can only be interpreted as predictors.

Strengths of this investigation are its large representative sample which was collected over five survey waves in ten consecutive months. Furthermore, we pre-registered a study and analysis plan before conducting the analysis.

## Conclusions

In conclusion, we were able to show that 7.0% of people in Germany who ever used cannabis have ever spoken with their GP, or received advice, about their cannabis use during a time when cannabis had not been legalised in Germany. These results offer a starting point to evalute the impact of the legalisation in the near future.

## Data Availability

The dataset used and analysed during the current study is available from the corresponding author on reasonable request.

## Abbreviations

GP: General practitioner
DEBRA: German Study on Tobacco Use
OECD: Organisation for Economic Co-operation and Development
DSM-IV: Diagnostic and Statistical Manual of Mental Disorders, fourth edition
